# Ten “simple” rules for non-Indigenous researchers engaging Indigenous communities in Arctic research

**DOI:** 10.1371/journal.pcbi.1012093

**Published:** 2024-06-27

**Authors:** Joy M. O’Brien, Nathan Blais, Carmen Butler, Natalie White, Ash Bustead, Collin Figler, McKenna Wells, George Anderson, Anna Yuhas, Jessica Gilman Ernakovich

**Affiliations:** 1 Department of Biology, Indiana University, Bloomington, Indiana, United States of America; 2 Department of Natural Resources and the Environment, University of New Hampshire, Durham, New Hampshire, United States of America; 3 Center of Soil Biogeochemistry and Microbial Ecology (Soil BioME), University of New Hampshire, Durham, New Hampshire, United States of America; Dassault Systemes BIOVIA, UNITED STATES

## Introduction

Climate change continues to disproportionately affect the Arctic, contributing to a pressing need for research in Arctic systems. As a result, more scientists are conducting fieldwork in Arctic regions, where Indigenous people have lived on lands and territories for time immemorial. This necessitates that the scientific community conduct research ethically and inclusively [1,2] given that research in the Arctic historically was (and often still is) based on colonial frameworks that are highly exclusive, extractive, and invasive, as well as physically and mentally damaging to generations of Arctic Indigenous people. For example, between 1955 and 1957, Dr. Kaare Rodahl administered radioactive iodine to Arctic Indigenous peoples without their knowledge to determine if they were physiologically adapted to cold conditions [3,4]. Other examples include using blood samples from Indigenous individuals for additional unconsented purposes, disrespect for Indigenous property, and blatant disregard for Indigenous peoples and their consent and advice [3,5,6]. In ecological research, misconduct on the part of the researcher may manifest in ways more subtle to the non-Indigenous researcher. Examples include, but are not limited to, taking credit for Indigenous knowledge and discoveries, neglecting to use original Indigenous names for organisms, places, and people, and failing to seek and receive approval to work on Indigenous lands [7,8]. Given the historical legacies between non-Indigenous scientists and Indigenous communities, Indigenous communities often remain cautious when interacting with researchers. Likewise, some researchers are hesitant to engage with Indigenous communities based on perceived challenges and unfamiliar cultural barriers [9], despite the growing availability of resources and guides developed by both Indigenous communities and non-Indigenous researchers regarding research collaborations [10–16]. It is likely that the history of unethical engagements and mistrust contribute to the underrepresentation of Indigenous researchers in STEM fields, and American Indians and Alaska Natives combined make up less than 1% of STEM professionals in the US [17,18]. The hesitancy of non-Indigenous scientists to approach Indigenous communities impedes progress towards addressing the pressing social, infrastructural, and environmental challenges associated with Arctic climate change that affect local Indigenous communities.

Despite claims of significant increases in transparent and participatory research in the Arctic, non-Indigenous Arctic researchers have made little progress on this front [2,19,20]. Pitseolak Pfeifer [10], the CEO of Qikiqtaaluk Corporation in Iqaluit, Canada writes “Arctic research continues to operate in a colonial framework and with an academic mindset that largely privileges the interests of southern [non-Arctic] institutions and fails to address Northern [Arctic] societal needs and issues, in particular, those experienced in Inuit communities [p.1].” Today, Arctic Indigenous communities continue to experience insensitive and disingenuous interactions with non-Indigenous researchers perpetuating historical trends [9] and inequitable power imbalances [19]. This needs to change; researchers must learn to ethically engage with Indigenous communities as they conduct research in ways that are empowering, reciprocal, and genuine. With this in mind, we acknowledge that this ethical engagement comes within the framework of the existing power structures that favor non-Indigenous society and ways of thinking, which shape relations between non-Indigenous and Indigenous peoples [21]. To engage ethically, progress must be made in dismantling the power imbalance between Indigenous and non-Indigenous cultures.

While it is important to engage with Indigenous communities for ethical reasons, these communities also hold valuable knowledge and skills that if ignored may lead to lower-quality research. Indigenous communities have detailed knowledge of their land, giving them the ability to expertly monitor wildlife and manage resources, among other practices [22]. For example, the Inuit from Mittimatalik on Baffin Island Nunavut, Canada helped non-Indigenous researchers expand their documented knowledge of the spatial and temporal ranges of Arctic fox [*Vulpes lagopus*] and Greater Snow Goose [*Chen caerulescens atlantica*] [23]. Indigenous knowledge and monitoring often extend temporally beyond non-Indigenous science because it spans centuries to millennia while non-Indigenous monitoring practices are limited to decades or shorter [24]. Additionally, Indigenous Arctic communities like the Inuvik from Ikaahuk, a coastal community within Sachs Harbour on the Beaufort Sea, have extensive and detailed knowledge of changing sea ice patterns as well as a high-resolution view of local environmental processes that are invaluable to decision-making in a changing Arctic [24].

**Fig 1:**
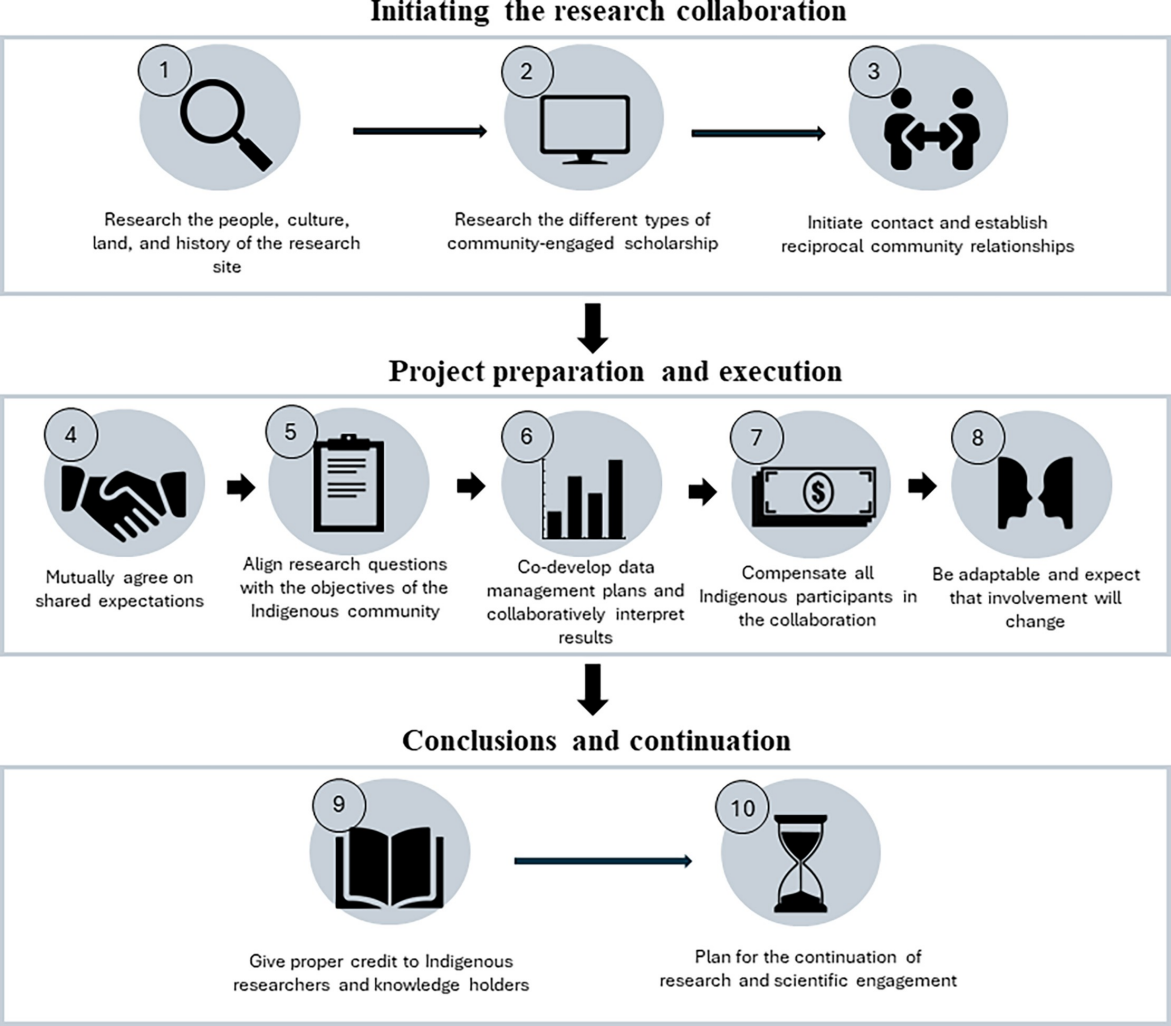
Flowchart of the 10 “simple rules” to guide research collaborations with Indigenous communities. These 10 rules are categorized into 3 stages: initiating the research collaboration, project preparation and execution, and conclusions and continuation of research. Rules within each stage may be implemented concurrently; although they are shown linearly for clarity, a linear progression between and within stages should not be expected as every collaboration is unique. Figure designed by Julia Saltzman, University of New Hampshire, Research and Large Center Development.

Here, we outline the best practices for non-Indigenous researchers to employ before, during (including pre-proposal activities through project execution), and after research collaborations with Arctic Indigenous communities. We chose to focus on Arctic regions in North America and Scandinavia (including the high, low, and subarctic) drawing examples from locations in Alaska, United States of America, Canada, and Finland. We acknowledge there are also Indigenous communities in other locations such as the Russian Arctic who may require unique considerations but are out of the geographical scope of this work due to lack of accessibility to these communities [25]. While there are several resources detailing the best practices for collaborating with Indigenous communities in the Arctic, this topic can appear overwhelming, which may discourage research engagement with Indigenous communities. Thus, we aimed to create a succinct summary of the main lessons from this literature to serve as a starting point for researchers. We offer our position in the form of “Ten Simple Rules” (Fig 1) taking care to acknowledge that these rules are not actually simple because they require not only building relationships, but also building those relationships across the spans of cultures, geographic distances, and incentive structures.

We acknowledge our positionality as non-Indigenous scholars from academic institutions within the contiguous United States. While we sought to utilize Indigenous-authored literature and other primary resources when available, this work primarily comes from the perspective of non-Indigenous scholars and therefore may not reflect Indigenous views unless directly quoted. However, since it is the responsibility of the researcher(s) to educate themselves on how to engage ethically with Indigenous communities, we believe we are well-positioned to collate these 10 “simple rules.” This work ideally provides a framework for scholars to engage ethically and authentically with Arctic Indigenous knowledge holders, while also helping to alleviate the burden on Indigenous scholars and communities, who are involuntarily tasked with educating non-Indigenous researchers on how to engage with their respective communities.

## Initiating the research collaboration

### Rule 1: Research the people, culture, land, and history of the research site

Researchers should prepare for Arctic research by familiarizing themselves with the culture of the Indigenous peoples they seek to collaborate with, as there are many Indigenous tribal nations whose customs, traditions, and history differ significantly. Reading current literature and white papers published by Indigenous scholars and communities is vital, as many have criticized the actions of current researcher engagement as superficial (e.g., Korthius and colleagues [9]). Good resources include but are not limited to tribal code and sovereignty documents [11]. Many of these highlight ways to maximize collaboration and minimize intrusive behavior in research practices by drawing from community representation programs and the co-development of research goals [12–14]. Additionally, accessing knowledge outside the written scholarship like performances, artistry, and storytelling, to name a few examples, can also provide valuable insight into the culture and perspectives of Arctic Indigenous people.

With this, non-Indigenous researchers must recognize the historical and continued impact of colonization on Arctic Indigenous communities, including the impact of extractive non-Indigenous science. Awareness of historical injustices and the effects of colonization will provide context to the interactions between researchers and Indigenous peoples, prompting researchers to evaluate the social consequences of their work [14]. Lack of preparation and knowledge on the part of the researcher regarding the impacts of colonization can limit the potential for community participatory research and engagement and further reinforce colonial norms [15]. A 2018 study revealed that only 50% of early career researchers feel equipped to properly engage with Arctic Indigenous communities, while 79% of all Arctic researchers would like to have higher levels of Indigenous community involvement [16]. Proper preparation enables researchers to confidently and effectively engage with the Indigenous community to foster a positive and respectful environment that promotes participation and beneficial research outcomes for both parties [26–28].

### Rule 2: Research the different types of community-engaged scholarship

Understanding and respecting different approaches to knowledge production can help to prepare the non-Indigenous researcher to work with Indigenous communities. This is crucial because previously employed research methodologies have traditionally been based on colonial ideology [29,30] with a racist underpinning that prioritizes non-Indigenous research institutions rather than Northern interests [10]. For example, “parachute research” or “helicopter research” is used to describe scenarios when researchers collect data and promptly leave, without acknowledging the Indigenous community, land, or resources affected [26,31]. It also explains scenarios when a researcher sets up a program for Indigenous engagement (e.g., speaking to local students) that occurs as a one-off. As a result, the word “research” has become a dirty word among many Indigenous groups [5,14,32]. To move away from harmful colonial research practices, there are a variety of community-based research and partnership approaches which include but are not limited to the Co-Production of Knowledge [19,33,34], Community-Based Participatory Research (CBPR) [5], and Citizen Science [35]. While the frameworks are highlighted individually in this section, they are not mutually exclusive, and researchers may find value in employing a combination of these approaches to foster an inclusive and equitable research environment.

**Co-Production of Knowledge (CPK)** is a framework that is defined by a partnership between an Indigenous community and non-Indigenous researchers [19,33,34]. CPK is analogous to cross-disciplinary research between 2 scientific fields where both forms of knowledge have equal value. In a co-production of knowledge research model, Indigenous ways of knowing and non-Indigenous scientific approaches have equal value and new knowledge is produced through cooperation to address a shared issue or goal [36]. Within a CPK framework, data collected through research should reflect the wants and needs of the Indigenous community, such as in the development of climate adaptation plans [21]. For example, thawing permafrost is causing severe damage to local infrastructure in the Arctic [37], a problem that is best addressed with methodologies and ways of knowing from a combination of engineers, community leaders, and scientists to build a holistic mitigation strategy.

**Community-Based Participatory Research (CBPR)** is joint research conducted by both communities and researchers [5]. Equitable involvement, respect, and collaboration are critical to effective CBPR which should be cultivated through trust building, education, and openness to differing world views [5,38]. In CBPR, community members and researchers come together as equals to discover and share knowledge. For example, research can be guided by a committee composed of council members, youth, Elders from the community, and researchers. CBPR has been heralded as a method that can aid the transfer of intergenerational knowledge [5].

**Citizen science** educational programs allow for community engagement and participation in the scientific process through collecting and analyzing data independently and/or alongside researchers [35]. Engaging with Indigenous communities in citizen science allows for the implementation of local and Indigenous knowledge into scientific practices, policy, and conservation [39]. For example, the “Arctic Salmon Initiative” based in remote regions of the Canadian Arctic, aimed to monitor salmon populations in response to environmental change through citizen science [40]. Indigenous and non-Indigenous citizens reported their salmon harvest and supplied samples to researchers which amounted to a more comprehensive understanding of salmon presence and movement in the Canadian Arctic. However, successful citizen science projects can be limited by the lack of Northern infrastructure such as unreliable internet and electricity, and resistance by local government (e.g., permission to access land and resources) [41]. The prepared researcher will contact the community including local governments before the onset of any citizen science programs (Rule 3) which will aid in the identification of potential challenges long before program implementation. The theme of preparation and early identification of challenges extends into other rules such as Rule 4 “Mutually agree on shared expectations” and Rule 5 “Align research questions with the objectives of the Indigenous community.”

These frameworks of community research allow space for Indigenous community members to operate as true collaborators—to take on positions that allow them to govern, design, and co-produce as much of the research as they are willing. Advancing Indigenous governance in research influences the trajectory of research and benefits the entire research process [15]. Greater active participation has the potential to lead to more meaningful relationships, as people are more likely to have a vested interest in the success of a study that they helped plan and execute [6].

### Rule 3: Initiate contact and establish reciprocal community relationships

Initiating meaningful, professional relationships can sometimes be a daunting, intimidating, and slow endeavor. However, just like the beginning of any other research collaboration, it is necessary to reach out to Indigenous communities to gauge their interest as potential collaborators well in advance of writing grants and proposals. Establishing trust with an Indigenous community requires time, patience, and consistent communication throughout the collaborative process [1,12,19]. For example, in a 2015 discussion between Dr. Kathi Wilson, a professor in the Department of Geography, Geomatics, and the Environment at the University of Toronto, and Mary Ellen Thomas, the Senior Science Advisor for Nunavut, Mary suggested that “[Indigenous] people(s) don’t really pay attention to you until at least the third visit ([15] p. 8).” Researchers should recognize that their agenda will not be a top priority regardless of academic timelines. They should work slowly to build relationships with a foundation of trust, looking toward shared experiences and common concerns to co-develop aims [15,19]. Consequently, approaching Indigenous communities after writing proposals or near deadlines for submission should be avoided. This implies that the relationship might be insincere, resembling a mere box-checking exercise which is disrespectful and unlikely to lead to a fruitful collaboration [9].

Relational accountability and mindful reciprocity are 2 key concepts to remember when it comes to building ethical relationships with Indigenous communities [5,15,33]. Relational accountability is the inherent responsibility of researchers to cultivate relationships, fulfilling their role as trusted scientists [42] and collaborators throughout the research process. Mindful reciprocity is characterized as a mutually beneficial exchange based on building relationships around shared humanity and common objectives, rather than the pursuit of research goals that could potentially exclude a community’s cultural significance [43]. Recognizing shared humanity helps to dismantle existing power dynamics that diminish Indigenous ways of life and autonomy [14,44–46].

Determining the community’s preferred contact method is a respectful first step in starting a working relationship. One appropriate method to initiate contact is to interface with Indigenous organizations, such as the National Representational Organization for the Inuit in Canada (Inuit Tapiriit Kanatami), which can help facilitate relationships between researchers and community members [47]. Likewise, if a preferred conduit for contact exists for a particular person or community, it must be utilized (cold emailing or calling should be avoided if alternatives are stated). For example, the Nunatsiavut Inuit community has a research advisory committee, a specific application portal, and guidelines for research proposals [47]. Additionally, researchers could seek introductions from close colleagues who have established ties to a particular Indigenous community, as this reinforces previously successful relationships where both parties were satisfied with the outcomes [21,26]. During initial contact, you may discover that your potential community collaborators have no desire to participate. While this outcome is not ideal for the researchers, it must be respected.

## Project preparation and execution

### Rule 4: Mutually agree on shared expectations

Respectfully and effectively collaborating with Arctic Indigenous communities requires established agreed-upon expectations for research engagement, involvement, and output [48,49]. Researchers should draw upon the knowledge gained from completing Rules 1 and 3 to co-create a written list of expectations that can be referred to throughout the project. In many cases, these will need to be approved by community governance structures. Additionally, it is important to respect and embrace the level of involvement the community desires in the development of agreements [50]. As with any partnership, reaching a consensus is important and should be given sufficient value (e.g., time in discussions), given that different perspectives and personalities are likely to cross during such a collaboration. This is particularly important because many Indigenous communities govern with a consensus model in contrast to a representative democracy model many non-Indigenous researchers may be accustomed to [51]. Cooperation and consensus between project participants are often best achieved when face-to-face interactions are prioritized [6].

The following are suggested topics that can spur conversation and promote equity when forming collaboration expectations and before establishing research priorities. Determine (1) the best methods and times for communication including but not limited to, meetings, outreach, educational materials, webcasts, emails, radio, and translations [16,52]; (2) the roles and responsibilities and the expected outcomes for each collaborating party [16]; (3) employment and or educational opportunities associated with the project [48,52,53]; (4) data sharing preferences, interpretation, and publication [11,16]; and (5) the research project timeline [2,52].

When conducting research that involves any human participation, it is necessary to investigate the ethics and confidentiality surrounding the research plans [11]. For non-Indigenous researchers, this often includes university institutional review boards (IRB). The Indigenous community’s leadership should thoroughly vet these documents before submission. The development of the shared expectations document can also come in the form of a code of conduct or an explanation of team values. For example, as part of the CPK methodology employed in the Kake climate partnership—a collaborative project between Indigenous and non-Indigenous partners in Southeast Alaska—Figus and colleagues [54] documented principles and expectations such as tribal values, food sovereignty, data sovereignty, and publication values.

### Rule 5: Align research questions with the objectives of the Indigenous community

One of the most important aspects of conducting research with an Indigenous community is to align the research goals with those of the community. Therefore, researchers should include Indigenous communities in the beginning stages of the research process [21,48]. Establishing a mutual understanding of research objectives and maximizing transparency prevents researchers from imposing on Indigenous peoples and avoids exploitative practices. The inclusion of diverse voices and different ways of knowing in the development of research questions facilitates a sustainable research project that is valuable for the Indigenous community and non-Indigenous researchers [19,54].

Despite the benefits, aligning research objectives with the community requires adaptability, flexibility, compromise, and communication on the researcher’s part [38,55]. To facilitate the alignment of objectives, researchers should host meetings or workshops with Indigenous organizations to directly engage with Elders and other community leaders [56]. For example, one successful collaboration studying the effects of severe weather and storm surges on coastal Alaskan towns began with presenting the project idea to the community, meeting with the tribal council, and holding one-on-one interviews with community members [57]. Collaborations are likely to be successful for projects where Indigenous communities benefit from the research. For example, Arctic change research can address issues relating to climate resilience, relocation, or food security [5,13]. But to successfully address these issues, both the researcher(s) and the community must communicate and create an action plan detailing the research question(s), objectives, and aims.

### Rule 6: Co-develop data management plans and collaboratively interpret results

Data should be accessible to the community because of their contribution to its collection, their ownership of the land from which the data was generated, and their ability to benefit from the findings. Researchers must conduct themselves ethically by ensuring Indigenous access, ownership, and control over data and information [6,15] through what is known as Indigenous data sovereignty (IDSov) [11]. This includes but is not limited to agreements regarding data ownership, availability, use, and interpretation. When producing shared data, researchers should abide by the CARE principles of data governance, which are a set of standards that outline the best practices for sharing and using data involving Indigenous communities and their property [11]. The CARE principles encompass collective benefit, authority to control, responsibility, and ethics [11]. Further, both original data and compiled results should be provided free of charge in a format agreed upon at the outset (whether this is an easily digestible brochure translated to the local language, a set of figures for a government report, or raw data) [29,33].

Disseminating compiled results to community members through public presentations, community newsletters, web platforms, and local media outlets allows more members of Indigenous communities to utilize the findings of the study [58], and sharing raw data with appropriate tribal agencies can allow Indigenous data to have an impact beyond the project period [11]. When sharing results, it is necessary to consider the needs of the target audience and to consult with the community about the dissemination of the study findings in the context of Indigenous objectives and sovereignty [59]. Indigenous collaborators, who are most equipped to elucidate the best method of sharing data and results with their community, should be consulted before disseminating the results within and beyond their community. Research findings should be framed in a context that is directly relevant to local issues and community needs, such as threats to subsistence hunting or community infrastructure [58]. Benefits to communities can be further achieved if findings are integrated into public development [26]. For example, information about rapid permafrost degradation could be used to better protect against impending climigration in an Arctic community [60]. By ensuring the results are accessible to the community and presented in the context of local challenges, researchers can enable the community to reap the full benefits of the research.

### Rule 7: Compensate all Indigenous participants in the collaboration

Indigenous collaborators should be considered equal contributors and compensated as formal members of the research team [2], as their contributions are essential to the quality and validity of the study. Compensation should be provided for all levels of participation including but not limited to the knowledge provided by Elders [53]. Types of participation requiring compensation include interviews or consultations, fieldwork, data collection, equipment maintenance, navigation, data entry, project development, co-production of research, and leadership. Financial compensation for participants should be included in the project budget as a default [2,48], with room and understanding that the form of compensation may change with Indigenous priorities. Compensation can be monetary, but compensation in the form of goods, transportation, educational experiences, and trading may be more advantageous in some situations or for some participants. Like other rules, this requires a conversation with the intended partners before project initiation. Appropriate compensation reinforces the value of the work and shows respect for the community [57].

### Rule 8: Be adaptable and expect that involvement will change

Arctic research with Indigenous collaboration requires a high degree of adaptability due to changing circumstances in response to weather events, cultural practices, and seasonal events (such as subsistence hunting) [57]. Priorities for the researcher may differ from the priorities of Indigenous peoples during the research process. Cultural practices may also trump research needs. For example, Eerkes-Medrano and colleagues detailed working with Alaska Indigenous communities when the project was put on hold for several days following the passing of a community member, which by custom halted all public events until after the funeral. Also, Arctic Indigenous communities rely on subsistence hunting and gathering not only to meet nutrition requirements but also for spiritual connection to the Earth. When favorable hunting or gathering conditions emerge, researchers might find their Indigenous contacts to be away without advanced notice, which can pose challenges to research timelines [57]. However, to remain respectful of their Indigenous partners, researchers must remain adaptable and recognize Indigenous cultural priorities by changing research objectives or by planning for absences and known setbacks in advance.

## Conclusions and continuation

### Rule 9: Give proper credit to Indigenous researchers and knowledge holders

When Indigenous peoples contribute their unique perspectives and approaches to experimental methods and design, the scope of the study is strengthened and therefore this effort should be properly recognized [2,6,10,52,61]. It is imperative to ask how Indigenous collaborators would like to be acknowledged, and if it is appropriate, they should be included in the co-authorship of research products [48,56,61]. Early initiation of conversation regarding co-authorship is key to addressing uncertainties and maintaining transparency [61]. Additionally, co-producing an authorship agreement can aid in this process and establish expectations in writing [61]. The CBPR framework for research incorporates this style of cooperation [5]. We recommend creating an authorship agreement that highlights the needs and wishes of the community, detailing whether the community wants to contribute to authorship as a whole, as individuals, or not at all. If a community or an individual does not wish to be identified for any reason, confidentiality must be upheld, which may mean not disclosing identifying information of individuals or the community [53,61]. While early conversations will hopefully reveal the wishes of the community (Rules 4 and 5), individuals and communities may alter their preferences throughout the research process, which should be expected. Lastly, while this rule is presented in the context of concluding research, it should be implemented in other stages of the collaboration when appropriate, such as writing grant proposals.

### Rule 10: Plan for the continuation of research and scientific engagement

Designing and executing research projects centered around community needs will most likely lead to situations where the project lasts longer than the funding. Starting discussions about the community’s desire for project longevity and future self-management is integral, especially during the initial phases of project preparation and execution (Fig 1). This is especially important given the short timeframes of funded research and field seasons [2,16], and the inevitable hurdles of maintaining continuous, long-term funding. Centering self-determination and capacity-building from the outset are project models that can lead to a natural continuation of the project aims (as demonstrated in [15]). Discussions should include planning for the resources and training that local project management will require. Citizen science and youth programs are demonstrated conduits for the transfer of leadership [6,15]. For example, the Alaska Beluga Whale Committee (ABWC)—a partnership between Alaska Native hunters, non-Indigenous scientists, and agency representatives to manage beluga whale populations—has endeavored to integrate TEK and non-Indigenous scientific approaches in the study design of whale management. To study the beluga populations, hunters and scientists participated in tagging, to complement previous hunter knowledge of whale tracking, which enables current and future self-determination regarding food sovereignty [6]. In another project, scientists, nonprofits, and the Akiak Native tribal council co-produced novel methods to harvest rainwater for consumption; the continuation of project aims was achieved through teacher training and the development of guidebooks [62]. However, simply maintaining contact via call, text, or email and acting as remote support can be impactful for ongoing efforts, as well as maintaining the human connection between Indigenous and research partners [63]. Facilitating the ability of Indigenous communities to sustain programs strengthens both the science and the relationships between Indigenous communities and non-Indigenous researchers [5].

## Conclusions

The purpose of this paper is to highlight guidelines and best practices for initiating and building research collaborations with Arctic Indigenous communities. In addition to these rules, we have included a table of resources that exemplify each rule to guide further detailed reading (S1 Table). As the ethos surrounding scientific engagement continues to change, we recognize that these rules are not all-encompassing and that they will continue to evolve; however, we hope they aid non-Indigenous researchers in collaborating ethically and responsibly with Indigenous communities. Following the prescriptions in these rules in their entirety is a major time commitment, which may be perceived as a barrier to conducting Arctic research; however, every step is vital to conducting ethical and impactful research with Indigenous peoples and their land and resources. This commitment is fundamental to advancing any community engagement in Arctic research, as it centers Indigenous sovereignty, as well as fosters empowerment, and self-determination.

## Supporting information

S1 Table List of readings that include useful examples or resources for each rule. (DOCX)
