# Supplementary material for: Ten “simple” rules for non-Indigenous researchers engaging Indigenous communities in Arctic research
Source: PLoS Comput Biol. 2024 Jun 27;20(6):e1012093. doi: 10.1371/journal.pcbi.1012093 (PMC11210771; doi:10.1371/journal.pcbi.1012093)
Supplement: S1 Table — (DOCX) [file pcbi.1012093.s001.docx]

| **Rule** | **Reference** | **Description** |
| --- | --- | --- |
| 1. Research the people, culture, land, and history of the research site | Tuck & Yang, 2012 [1] | Defines the meaning of decolonization and describes settler colonial frameworks (full text) |
| 2. Research the different types of community-engaged scholarship | Herman‐Mercer et al., 2023 [2]  Tobias et al., 2013 [3]  Dunmall & Reist, 2018 [4] | Co-production of knowledge (CPK) collaboration, see section 3.2 “Working With Communities: Developing Knowledge Co-Production Protocols” p.8  Community-based participatory research (CBPR) collaboration (full text)  Citizen science collaboration monitoring salmon in the Arctic, see the “Methods” p.34 |
| 3. Initiate contact and establish reciprocal community relationships | Nunatsiavut Government Research Advisory Committee <https://nunatsiavutresearchcentre.com/ngrac/> [5] | Nunatsiavut Inuit community research advisory committee application portal and guidelines for research proposals |
| 4. Mutually agree on shared expectations | Figus et al., 2022 [6] | See section “Building a partnership through written and agreed upon principles and expectations” section” p.7 |
| 5. Align research questions with the objectives of the Indigenous community | Pearce et al., 2009 [7] | See section “Community involvement in research design and development” p.19 |
| 6. Co-develop data management and collaboratively interpret results | Carroll et al., 2022 [8] | Outlines and describes CARE principles, see tables 1 & 2 and the section “Discussions and Recommendations” p.7 |
| 7. Compensate all Indigenous participants in the collaboration | Doering et al., 2022 [9] | Describes proper funding structure when working with Indigenous communities/collaborations,  see section 5 “How can funding agencies enable and support meaningful collaboration and co-creation?” p. 5 |
| 8. Be adaptable and expect that involvement will change | Eerkes-Medrano et al., 2019 [10] | Describes challenges during site visits and gives context for the importance of adapting/being flexible during the research process, see the section “Challenges during Site Visits” p.17 |
| 9. Give proper credit to Indigenous researchers and knowledge holders | Castleden et al., 2010 [11] | Outlines authorship practices with Indigenous collaborators as well as the benefits and risks (full text) |
| 10. Plan for the continuation of research and scientific engagement | Wilson et al., 2020 12] | Lays out the Sikumiut model for self-determination, see section “From guidance to practice: the Sikumiut model” p.134 |

**S1 Table**

**References**

1. Tuck E, and K. Wayne Yang. “Decolonization Is Not a Metaphor.” Decolonization: Indigeneity, Education, & Society 1, no. 1 (2012): 1–40.
2. Herman‐Mercer N, Andre A, Buschman V, Blaskey D, Brooks C, Cheng Y, et al. The Arctic Rivers Project: Using an Equitable Co‐Production Framework for Integrating Meaningful Community Engagement and Science to Understand Climate Impacts. Community Sci. 2023;2(4):e2022CSJ000024.
3. Tobias J, Richmond C, Luginaah I. Community-Based Participatory Research (CBPR) With Indigenous Communities: Producing Respectful And Reciprocal Research. J Empir Res Hum Res ethics. 2013;8(2):129–140.
4. Dunmall, KM, and JD Reist. “Developing a citizen science framework for the Arctic using the ‘Arctic Salmon’ initiative.” *Impacts of a Changing Environment on the Dynamics of High-Latitude Fish and Fisheries*, 2018, pp. 31–47, https://doi.org/10.4027/icedhlff.2018.02.
5. “Nunatsiavut Government Research Advisory Committee.” *Nunatsiavut Research Centre*, 14 Sept. 2019, nunatsiavutresearchcentre.com/ngrac/.
6. Figus E, Ki’yee Jackson B, Trainor SF. The Kake Climate Partnership: Implementing a knowledge co-production framework to provide climate services in Southeast Alaska. Front Clim. 2022;4.
7. Pearce TD, Ford JD, Laidler GJ, Smit B, Duerden F, Allarut M, et al. Community collaboration and climate change research in the Canadian Arctic. Polar Res. 2009;28(1):10–27.
8. Carroll SR, Garba I, Plevel R, Small-Rodriguez D, Hiratsuka VY, Hudson M, et al. Using Indigenous Standards to Implement the CARE Principles: Setting Expectations through Tribal Research Codes. Front Genet. 2022;13(March):1–10.
9. Doering N, Dudeck S, Elverum S, Fisher C, Henriksen JE, Herrmann TM, et al. Improving the relationships between Indigenous rights holders and researchers in the Arctic: An invitation for change in funding and collaboration. Environ Res Lett. 2022;17(6).
10. Eerkes-Medrano L, Huntington HP, Castro AO, Atkinson DE. Engaging Northern Indigenous Communities in Biophysical Research. Arctic. 2019;72(2):166–80.
11. Castleden H, Morgan VS, Neimanis A. Researchers’ perspectives on collective/community co-authorship in community-based participatory Indigenous research. J Empir Res Hum Res Ethics. 2010 Dec;5(4):23–32.
12. Wilson KJ, Bell T, Arreak A, Koonoo B, Angnatsiak D, Ljubicic GJ. Changing the role of non-indigenous research partners in practice to support inuit self-determination in research. Arct Sci. 2020;6(3):127–53.
